# The Development of an Age-Structured Model for Trachoma Transmission Dynamics, Pathogenesis and Control

**DOI:** 10.1371/journal.pntd.0000462

**Published:** 2009-06-16

**Authors:** Manoj Gambhir, Maria-Gloria Basáñez, Matthew J. Burton, Anthony W. Solomon, Robin L. Bailey, Martin J. Holland, Isobel M. Blake, Christl A. Donnelly, Ibrahim Jabr, David C. Mabey, Nicholas C. Grassly

**Affiliations:** 1 Department of Infectious Disease Epidemiology, Imperial College London, London, United Kingdom; 2 International Centre for Eye Health, London School of Hygiene & Tropical Medicine, London, United Kingdom; 3 Clinical Research Unit, Department of Infectious and Tropical Diseases, London School of Hygiene & Tropical Medicine, London, United Kingdom; 4 Viral Disease Programme, MRC laboratories, Fajara, Banjul, The Gambia; 5 MRC Centre for Outbreak Analysis and Modelling, Department of Infectious Disease Epidemiology, Imperial College London, London, United Kingdom; 6 International Trachoma Initiative, New York, New York, United States of America; University of California San Francisco, United States of America

## Abstract

**Background:**

Trachoma, the worldwide leading infectious cause of blindness, is due to repeated conjunctival infection with *Chlamydia trachomatis*. The effects of control interventions on population levels of infection and active disease can be promptly measured, but the effects on severe ocular sequelae require long-term monitoring. We present an age-structured mathematical model of trachoma transmission and disease to predict the impact of interventions on the prevalence of blinding trachoma.

**Methodology/Principal Findings:**

The model is based on the concept of multiple reinfections leading to progressive conjunctival scarring, trichiasis, corneal opacity and blindness. It also includes aspects of trachoma natural history, such as an increasing rate of recovery from infection and a decreasing chlamydial load with subsequent infections that depend upon a (presumed) acquired immunity that clears infection with age more rapidly. Parameters were estimated using maximum likelihood by fitting the model to pre-control infection prevalence data from hypo-, meso- and hyperendemic communities from The Gambia and Tanzania. The model reproduces key features of trachoma epidemiology: 1) the age-profile of infection prevalence, which increases to a peak at very young ages and declines at older ages; 2) a shift in this prevalence peak, toward younger ages in higher force of infection environments; 3) a raised overall profile of infection prevalence with higher force of infection; and 4) a rising profile, with age, of the prevalence of the ensuing severe sequelae (trachomatous scarring, trichiasis), as well as estimates of the number of infections that need to occur before these sequelae appear.

**Conclusions/Significance:**

We present a framework that is sufficiently comprehensive to examine the outcomes of the A (antibiotic) component of the SAFE strategy on disease. The suitability of the model for representing population-level patterns of infection and disease sequelae is discussed in view of the individual processes leading to these patterns.

## Author Summary

Trachoma is the worldwide leading infectious cause of blindness and is due to repeated conjunctival infection with *Chlamydia trachomatis* bacteria. The effects of control interventions on population levels of infection and active disease can be promptly measured, but the effects on severe ocular disease outcomes require long-term monitoring. We present a mathematical model of trachoma transmission and disease to predict the impact of interventions on blinding trachoma. The model is based on the concept of multiple re-infections leading to progressive scarring of the eye and the potentially blinding disease sequelae. It includes aspects of trachoma natural history such as an increasing rate of recovery from infection, and a decreasing chlamydial load with subsequent infections. The model reproduces key features of trachoma epidemiology such as the age-profile of infection prevalence; a shift in the prevalence peak toward younger ages in higher-transmission environments; and a rising profile of the prevalence of the severe sequelae (scarring, trichiasis), as well as estimates of the number of infections experienced before these sequelae appear. The model can be used to examine the outcomes of various control strategies on infection and disease and can help to plan treatment interventions for different endemic settings.

## Introduction

Trachoma is the leading infectious cause of blindness in the world; 8 million people are blind or severely visually impaired due to trachoma and 63 million have active disease [1]. It is due to repeated conjunctival infection with the bacterium *Chlamydia trachomatis*, and the “SAFE” control strategy (surgery, antibiotics, facial cleanliness and environmental improvement) is recommended by the World Health Organization (WHO) [2]. The effects of control programs on community infection and active disease can be rapidly measured, but their effects on the severe sequelae (trichiasis, corneal opacity and blindness) will not be properly ascertained until decades after their implementation, so mathematical modeling provides an invaluable method for the prediction of program performance.

Previous mathematical models of trachoma infection at the population level have primarily looked at the effects of treatment with antibiotics, and the rebound in the prevalence of active disease that follows treatment cessation [3]–[6]. However, no model has taken into account the important effects upon infection and disease of the apparent increase in the rate of bacterial clearance that is observed with age. On the one hand, this shortening of clearance time is thought to be attributable to an acquired, yet not protective, immune response that is enhanced with repeated exposure to *C. trachomatis* [3],[7]. On the other hand, the mechanisms responsible for bacterial clearance may also be immunopathological, and they may lead to scarring and subsequent disease sequelae that are associated with trachoma [8]. Therefore, episodes of repeated infection and clearance of *C. trachomatis* may lead to the more severe complications of the disease: trachomatous scarring (TS), trachomatous trichiasis (TT), corneal opacity (CO) and, eventually, blindness. In order to predict the impact of treatment on scarring sequelae, which are the focus of the Alliance for the Global Elimination of blinding Trachoma by 2020 (GET 2020) [9], models need to incorporate contemporary understanding of the relationship between infection, disease, and disease progression.

A mathematical model of ocular infection with *C. trachomatis* was developed, and its parameters were estimated through fitting the model to pre-intervention ocular chlamydial infection prevalence, rate of recovery from infection, and infection load data from three geographically-separate study sites in The Gambia and Tanzania, representing areas of low, moderate and high endemicity. Insights from the model help explain observed age-profile patterns of infection prevalence in these settings. Progression of individuals to greater numbers of infections, through repeated infection, is interpreted as leading to worsening scarring. Therefore, the model population that has progressed to various numbers of repeat infection represents the population suffering from each of the severe disease sequelae; the corresponding age-profiles of disease prevalence are then compared with clinico-epidemiological data. Finally, the degree to which the model captures the epidemiological patterns of infection and disease observed, and the possible causes for discrepancy are discussed.

## Methods

### Model Development

**Figure 1:**
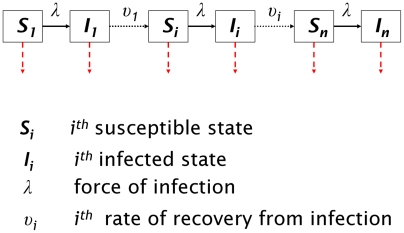
Compartmental diagram of the model. A compartmental diagram illustrating the model described in the text. Each susceptible and infected compartment is connected to the compartment above so that the population passes up a ‘ladder’ of infection. The subscript *i* corresponds to the number of prior infections experienced.

The model developed here represents ocular infection with *C. trachomatis* in a community setting and is based upon a framework commonly used in the modeling of microparasitic infections [10]. In the model, susceptible (*S*) individuals become infected through contact with infected (*I*) individuals before recovering again to a susceptible state. Initial infection and reinfection occur through direct contact with other infected members of the community whereas indirect contact can occur through inanimate objects capable of carrying infection from an infected person to another person or through facial contact with flies carrying the bacteria [11],[12]. In endemic settings, disease progression appears to occur through multiple reinfection [13],[14]. Therefore, the model takes account of the importance of multiple reinfections on disease progression by keeping track of the number of infections an individual has experienced. Superinfection of an already-infected individual with a different strain of *C. trachomatis*, which does occur in endemic villages [15], is ignored at this stage. Conceptually, the model represents a ‘ladder’ of infection, with each ‘rung’ of the infection ladder corresponding to an additional cumulative infection with *C. trachomatis* (Figure 1). Susceptible states are denoted by 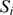 and infected states by 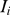, with subscript *i* denoting the number of previous infections experienced (full details of the model are given in Text S1). As individuals progress to the next state up the ladder of infection, a memory of the number of infections experienced is retained.

Since birth and death rates are important when determining prevalence levels of the more severe disease sequelae, the demography of the population is included in the model. The disease sequelae are more prevalent at older ages and, once the population has had the antibiotic component of the SAFE strategy successfully implemented, we assume that the rate at which disease prevalence levels decline depends upon mortality among older individuals. Age-specific death rates and the crude birth rate for The Gambia and Tanzania were based on WHO life table estimates for the year 2001 [16].

It is the explicit inclusion of disease sequelae, age structure, differential infectivity and immunity considerations that distinguish this model from those that have been previously reported [5],[6]. The equilibria of the model will provide a representation of the way in which each of these forces are balanced in the endemic state.

### Rate of Recovery and Infection Load

Several studies have postulated that the sequelae of trachoma are caused by immunopathological processes that increase in severity with increasing age [17]–[19]. This idea is supported by work which shows that the duration of episodes of infection and active disease (the latter encompassing trachomatous follicular and severe papillary conjunctivitis) becomes markedly shorter with increasing age [3],[7]. In this paper, it is assumed that adaptive immunity does not protect from acquiring infection but results in an increasing rate of recovery from infection as the number of previous infections increases; such a framework was chosen here due to the limited evidence for protective immunity against infection [20] and the preference for a parsimonious model. In Figure 1 the recovery rate from infection *I_i_* is denoted by 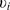. This recovery rate approaches a limit at high numbers of infections. The parameter values determining: 1) the rate at which the curve rises with infection number; 2) the initial recovery rate; and 3) the recovery rate following a large number of infections, were estimated using maximum likelihood by fitting the model to data on the prevalence of infection as detailed in the Text S1.

In trachoma-endemic communities, bacterial infection load among individuals at young ages is higher than that at older ages [21]–[23]. In the model presented here, this decrease in infection load with age is ascribed to the acquired immune response to chlamydial infection that is developed through bacterial reinfection. Text S1 describes the decay function that was used to represent the average infection load for an individual who has experienced a given number of infections. The chlamydial load enters the model as a proxy for the infectivity of individuals; those who have experienced fewer infections have a higher infection load than those who have experienced many and are therefore more infectious.

### Prevalence of Disease Sequelae

**Figure 2:**
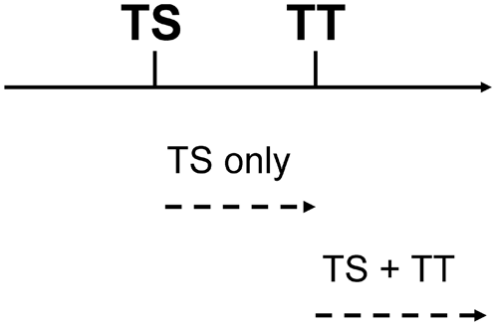
Incorporating disease sequelae into the model. A schematic representation of the way in which the model determines the presence of disease sequelae: trachomatous scarring (TS) and trachomatous trichiasis (TT) (TS and TT—but not corneal opacity, CO—are considered to be caused exclusively by reinfection with *C. trachomatis*). The simplest scheme is used: thresholds exist, along the ladder of infection, beyond which each of the sequelae are assumed to be present. Beyond the threshold corresponding to a specific sequela, that sequela is assumed to be observed, e.g. when the threshold for TT has been reached, both TT and TS are observed.

The model assumes that scarring worsens through repeat infection with *C. trachomatis* and that, as scarring (TS) becomes worse, the more severe disease sequelae (TT and CO) occur. However, due to the complex etiology of CO—reinfection is almost certainly not the only causal factor—only TS and TT are considered in the model, which is entirely based on the reinfection route. It is assumed that these conditions co-occur, so that an individual may have scarring, or scarring and trichiasis. In each case, it is assumed that where TT is present, TS will also be present (Figure 2).

### Infection and Disease Prevalence

The model was fitted using maximum likelihood to pre-intervention prevalence and chlamydial load data collected in studies carried out in The Gambia and Tanzania, the details of which have been described by Burton *et al.* [22], Solomon *et al.* [21],[24], and West *et al.* [25] (Table 1). In these studies community infection prevalence was determined by qualitative PCR and the individual infection load by quantitative PCR (in the case of the hyperendemic Tanzanian community, a model fit was also obtained for which quantitative PCR was used to measure both prevalence and load; further details of these are available in Text S1). These community data-sets illustrate the general differences between three distinct endemic levels while not necessarily being representative of all hyper-, meso- and hypoendemic communities.

**Table 1:** Summary of the infection and disease levels for the data-sets used in this study.

Location and country	Infection prevalence	Active disease prevalence	Source	Endemicity level
Upper Saloum district, The Gambia	7%	8%	[22]	Hypoendemic (<10% active disease)
Rombo district, Tanzania	10%	18%	[24]	Mesoendemic (10–20% active disease)
Kongwa district, Tanzania	52%	36%	[25]	Hyperendemic (>20% active disease)

Provenance of the data from three trachoma-endemic regions that are used in this paper for model fitting. Active disease is measured as trachoma follicular (TF) and/or trachoma inflammation (TI) on the World Health Organization simplified grading scheme [43].

The model was first fitted using maximum likelihood to the hyperendemic data set for which three distinct data types were available: age-profiles of the prevalence of infection and the infection load from a community in Tanzania [25], and the rate of recovery from infection based on a cohort study with frequent follow up in The Gambia [3]. A likelihood expression was formulated that combined all three data types and the prevalence, infection load, and duration of infection data were assumed to arise from binomial, Poisson and exponential distributions respectively. The Poisson distribution was selected in the absence of information regarding the distribution of the infection load for each infection category *i*; and an exponential distribution was used for the duration of infection data consistent with the model structure. The likelihood framework is outlined in Text S1. Likelihood expressions were formulated for each of the data sets and the overall log-likelihood (LL) formed by summing the individual LLs—and assuming that measurements of prevalence and load were independent of one another (Text S1)—so that:
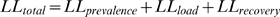
(1)The overall LL was maximized with respect to the six model parameters (listed in Table 2) pertaining to the three data types. Parameter values and their 95% confidence intervals (found using the profile-likelihood around the maximum *LL* value) are provided in Table 2. For each parameter, the profile likelihood was calculated by fixing the parameter and maximizing the *LL* with respect to all of the other parameters. Aside from the transmission parameter (*β*), the parameter estimates thus obtained were then used in modeling the meso- and hypoendemic settings (defined as those exhibiting an active disease prevalence lower than 10% and between 10 and 20% respectively (Table 1)) since they pertain to the biology and not to the transmission environment of the infection. Transmission parameters were then obtained separately for the hypo- and mesoendemic datasets based on maximum likelihood fitting to the prevalence data alone from each area. The pattern of population mixing among ages was assumed to lie between the extremes of entirely random and fully assortative [26]. The model was implemented in Matlab using the Euler integration method; the *LL* maximization was also performed using the Matlab package.

**Table 2:** Model parameter definitions and estimates.

Parameter	Parameter definition	Maximum likelihood estimate [95%CI] and units
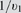	Mean duration of first infection	15.1 [6.5,23.3] months
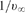	Mean duration of infection after multiple prior infections	2.8 [2.4, 3.2] months
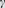	Rate of drop of duration of infection per prior infection	0.7 [0.1,∞] infection^−1^
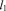	Infection load per person at first infection	1.0×10^5^[0.9, 1.3×10^5^] copies *C.trachomatis omp1* gene per ocular swab
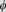	Rate of drop of infection load per prior infection	0.05 [0.03, 0.07] infection^−1^
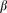	Transmission coefficient: the rate of transmission (per year) of infection between individuals	Hyperendemic: 27.7 [21.8, 35.1] year^−1^
		Mesoendemic: 2.4 [2.0, 2.9] year^−1^
		Hypoendemic: 1.8 [1.6, 2.1] year^−1^

Parameter definitions and estimates, with 95% confidence intervals, for the model obtained through maximum likelihood fitting using a function combining infection prevalence, bacterial load, and recovery rate data from a hyperendemic setting in Tanzania. The transmission parameters for the meso- and hypoendemic settings were estimated by maximum likelihood by fitting to prevalence data only. The parameter symbols refer to the model definition detailed in Text S1.

## Results

### Model Fit to Age-Profiles of Prevalence of *C. trachomatis* Infection by Endemicity Level

**Figure 3:**
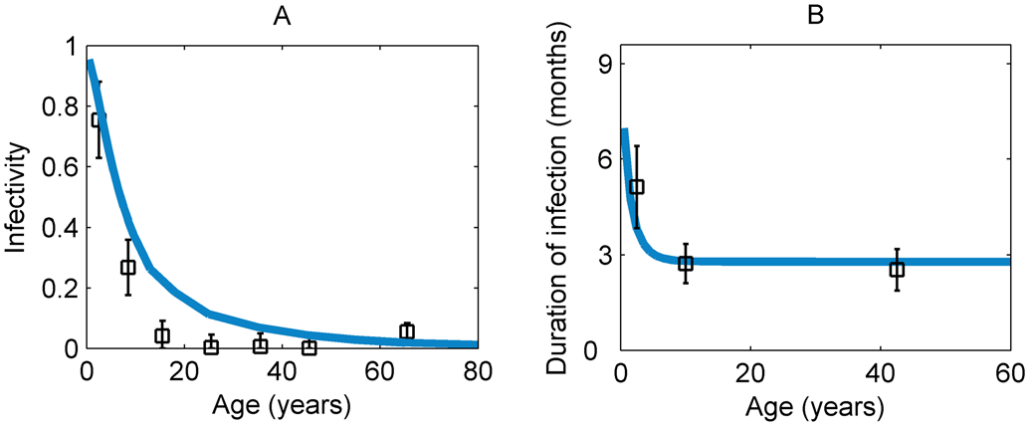
Model fits to hyperendemic data. Age-profiles generated by the maximum likelihood parameter estimates to the hyperendemic data set of West *et al.* [25] and—for the recovery rate—Bailey *et al.* [7], re-analyzed by Grassly *et al.* [3] (data displayed as square data points with 95% confidence intervals, and model fits shown as solid lines): A) the infectivity, which is proportional to the bacterial load, measured by quantitative PCR; B) duration of infection.

**Figure 4:**
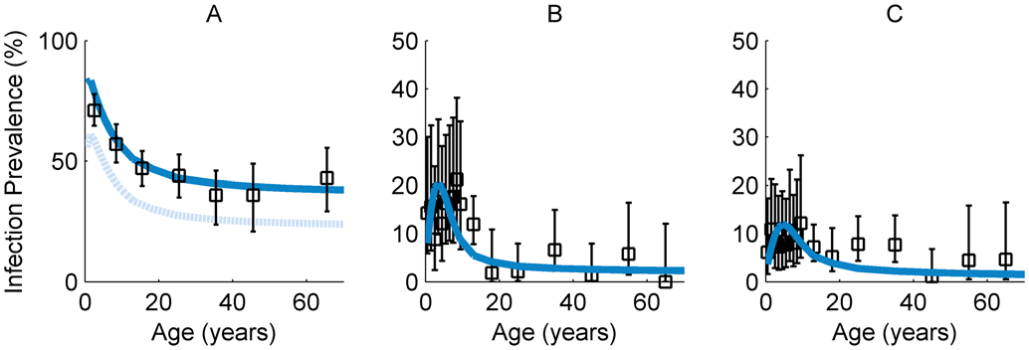
Model fits to data from three endemic settings. Age-profiles of the prevalence of infection generated by fitting the model (solid lines) to the data (squares and 95% confidence interval error bars) from (A) Kongwa, Tanzania [25] (B) Rombo, Tanzania [24] and (C) Upper Saloum, The Gambia [22]. The dotted line in (A) corresponds to a model-fit to the data from Kongwa, Tanzania assuming a lower number of true infection positives, namely those that were determined to harbour chlamydial loads as measured by quantitative PCR (see main text and Text S1). Note the different scale on the y-axis of (A) in comparison to (B) and (C).

The curves shown in Figure 3A and 3B represent the model-generated age-profiles of the infection load and the rate of recovery from infection based on the maximum likelihood parameters obtained from the analysis of data from the hyperendemic setting (*i.e.* as in Table 2). These curves show the previously described reduction in infection duration and intensity with age and the parameters thus obtained were used for all subsequent modeling. Model fits to the data by endemicity level are shown in Figure 4. The solid line in Figure 4A illustrates the model-generated infection prevalence curve corresponding to the fit to the hyperendemic data set published by West *et al.* [25]. The dotted line in Figure 4A is an illustrative fit obtained assuming a lower number of individuals in each age-group classified as positive for infection (see Text S1 for details). The transmission parameter *β* estimated for this adjusted dataset is approximately two thirds the size of the value shown in Table 2. Figure 4B and 4C illustrate the model-generated infection prevalence curves for the model fitted to the hypo- and mesoendemic datasets (*i.e.* using the endemic-specific transmission parameter estimate but the infectivity and rate of recovery parameter estimates obtained from the analysis of the data from the hyperendemic setting). Figure 3B shows that the duration of chlamydial infection declines from its initial maximum value to its plateau very rapidly with age, and this is due to the rapid decrease in this value with infection number.

The infection prevalence data come to a peak at young ages (roughly 5 years) in the hypo- and mesoendemic areas examined here, with model fits mirroring such peaks. Furthermore, the data also show some evidence for a peak shift [27], characterized by the peak of infection being shifted towards younger ages as transmission levels increase (Figure 4).

### Threshold Number of Infections for the Manifestation of Disease Sequelae

**Figure 5:**
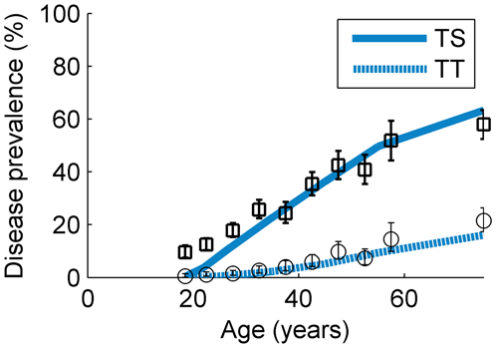
The age-dependent prevalence of the disease sequelae in a hyperendemic setting. Prevalence curves for trachomatous scarring (solid line) and trachomatous trichiasis (dotted line) are shown along with the data from [28] (circles (TS) and squares (TT) and 95%CI error bars), which were collected from the same district as the data of West *et al.* [25] during the mid-1990s, prior to drug treatment interventions. An assumption made here is that the incidence of the sequelae did not change over the decade prior to the first mass drug administration.

The threshold numbers of infections necessary for individuals to show signs of each of the sequelae were calculated for the hyperendemic setting. These thresholds were estimated by maximum likelihood using the published data of Munoz *et al.* [28], for the age-dependent prevalence of each of the disease sequelae. The maximum likelihood estimate for the threshold number of infections required for TS was 102 and for TT it was 151. In terms of the natural history of trachoma infection, disease, and disease sequelae, it is assumed here that these threshold values do not vary over the different endemicity levels but should be reached at different ages according to the intensity of transmission—individuals living in areas of different endemicity are assumed to show signs of each of the disease sequelae after having experienced the same number of infections, but they experience the sequelae *earlier in their lives* in those environments in which they are infected more frequently. The threshold infection numbers estimated here were used to generate the curves shown in Figure 5.

## Discussion

The model presented in this paper reproduces many important aspects of trachoma epidemiology, namely: 1) the pattern of the prevalence of infection with age, which peaks at very young ages and then declines; 2) a peak shift towards younger ages in this prevalence in higher transmission settings; 3) a rise of the prevalence level with higher transmission; and 4) a rise with age of the prevalence of the severe disease sequelae. The model also allows estimates to be made of the threshold number of infections necessary for the appearance of the severe disease sequelae. Infection and disease profiles were obtained assuming long-term stability of prevalence levels in the model, and therefore represent the equilibrium, pre-intervention state in each of the endemicity settings. However, a limitation of the data used to estimate model parameters is that in some areas (*e.g.*, The Gambia), there have been secular trends towards a decrease of trachoma incidence in the absence of control interventions. These trends are deemed much less significant in the hyperendemic setting.

Following the fitting of the model to the data-sets from the three endemic settings, the prevalence curves generated show a close correspondence with the trend in the observed profiles of infection prevalence with age. While good visual fits to the data are encouraging, a full analysis of the uncertainty in the parameter estimates is essential to judge how well-determined the model fits are. In the younger ages, the prevalence peak is caused by the long duration of infection, high chlamydial loads and intense transmission that result from patterns of assortative mixing by age. Subsequently, the prevalence of infection drops at older ages, as a consequence of the age-associated increase in the recovery rate from infection and the drop in infectivity with age: an individual who experiences an increasing number of infections recovers faster from each infection, with accompanying reductions in chlamydial load and infectivity. (In the model, the number of infections previously experienced tracks closely the age of an individual.) There is also a peak shift of the maximum infection prevalence towards younger ages (slightly greater than 5 years of age in the hypoendemic; slightly under 5 years in the mesoendemic, and very low (under 1 year) in the hyperendemic areas). For infectious diseases in general, this effect is usually due to acquired immunity and it occurs when individuals experiencing higher forces of infection either develop adaptive protective immunity earlier or clear their infection more rapidly at younger ages than they would in environments with lower force of infection. The observation of this phenomenon here lends further support to the importance of acquired immunity in trachoma [27],[29].

The recovery rate from infection rises very rapidly with the number of prior infections and the 95% confidence interval of the rate of this rise includes extremely large values at the upper end. This rapidity suggests that the immune response to the first few infections is qualitatively different to that of the bulk of subsequent infections and therefore the maturation of trachoma immunity occurs after only few infections, a finding that may also be associated with limited variation in the pathogen population. Indeed, the possibility of extremely large values for the rate at which the duration of infection changes with the number of infections (unbounded upper confidence limit of the rate 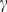, in Table 2), suggests that the data are consistent with the development of immunity following a single initial infection. The data for the recovery rate from infection (plotted in Figure 3B as its reciprocal, the average duration of infection) used in the model were on average lower than the estimates reported by Bailey *et al.* [7]—who used a test for infection less sensitive than PCR-based testing and may have found longer durations (*i.e.* lower recovery rates) with a more sensitive test—and instead corresponded with a newer analysis of the same data [3] (where the mean duration of infection was found to be around 5 months for young children (<5 years old) and under 3 months for older people (>15 years old)). If the rates of recovery in the model were not as low as those used in this work, the peak in infection prevalence, for settings with lower endemicity, would not occur at ages corresponding to those observed in the data. In the hyperendemic setting, the peak prevalence occurs at a very early age becoming barely perceptible due to its large transmission rate; this causes individuals rapidly to acquire infections from a very young age.

Infection bacterial loads are explicitly included in the model; the chlamydial loads for those who have experienced few infections are typically higher than for those who have experienced many. The reason behind this difference is thought to be the development of acquired immunity through repeated exposure to the bacteria that, although it does not protect from incoming infection, may reduce its intensity.

A model structure in which pathogen load is explicitly accounted for has been used extensively to model helminth infections, by assuming that acquired immunity to infection may be developed with cumulative infection experience and therefore with age, leading to peaked age-profiles of infection intensity and prevalence [29]–[31]. Age-specific changes in exposure are also likely to contribute to this pattern [32],[33], and indeed the peaked distributions observed in the model and the data for ocular chlamydial infection and disease are the result of both changes in the duration of infection and patterns of exposure to infection with age.

The prevalence levels of the disease sequelae were modeled under the assumption that individuals who had experienced greater than or equal to a specific threshold number of infections would begin to show signs of the ocular sequelae. Threshold infection numbers were therefore estimated corresponding to each of TS and TT; these calculations were performed for the hyperendemic setting, because it would only be in communities where there has been no intervention (at true endemic equilibrium) that the transmission and repeat infection rates will give rise to current disease sequelae prevalence levels. The threshold infection numbers for TS and TT estimated in this paper are dependent upon the data we have used for the duration of infection and infection load; a higher duration, for example, would decrease these estimates and so these values are contingent upon future longitudinal studies. In those communities (the hypo- and mesoendemic areas in this paper) where there has been either some intervention or possibly a secular trend that has reduced transmission, the prevalence of those suffering from sequelae will, for some time, remain much higher than the current transmission level would suggest. Another explanation for differences here is the possibility that only a given fraction of the population progresses to each of the disease sequelae and this fraction may vary between populations due to factors such as the genetic predisposition to scarring of particular individuals in each population [34]–[41].

Although our working hypothesis is that repeat chlamydial infection is the main route to the severe disease sequelae, it may not be the only one. Some studies show that, once established, scarring complications may continue to progress, perhaps driven by factors other than *Chlamydia spp.*, such as non-chlamydial bacterial infection [42]. Trachomatous CO leading to blindness probably has a multi-factorial etiology. These effects will be examined in future work and may lead to lower threshold infection numbers than those calculated here.

In summary, the balance between ocular exposure to *C. trachomatis* and acquired immunity, which is presumed to reduce the intensity and duration of infection, leads to the expected shape and magnitude of the age-profiles of infection prevalence observed in settings of variable endemicity. Data used in the model for the recovery rate from infection [3] led to lower corresponding parameter estimates than those previously reported [7], closer to those used in other trachoma models [5], although these models do not allow for an age-dependent recovery rate, nor do they investigate the relationship between infection and disease or incorporate chlamydial load. For the (hyperendemic) setting in which levels of current infection are those responsible for observed morbidity, the model captures well the progression of scarring with age and reproduces the observed age-profiles of ocular sequelae prevalence. Future work will investigate the effect of the ‘A’ component of the SAFE strategy (mass administration of antibiotics) on the age-profiles of infection and disease, and will present the implications of this model for trachoma control policy.

## Supporting Information

Text S1 Mathematical model outline and details on the likelihood.
(0.14 MB DOC)
