# Supplementary material for: The Development of an Age-Structured Model for Trachoma Transmission Dynamics, Pathogenesis and Control
Source: PLoS Negl Trop Dis. 2009 Jun 16;3(6):e462. doi: 10.1371/journal.pntd.0000462 (PMC2691478; doi:10.1371/journal.pntd.0000462)
Supplement: Text S1 — Mathematical model outline and details on the likelihood. (0.14 MB DOC) [file pntd.0000462.s001.doc]

**Supporting Information**

The model of trachoma transmission

The model that we have built for this study is made up from repeated susceptible and infected compartments, each given an index *i*. As individuals become infected and recover from infection, they move up a ‘ladder’ of infection through susceptible ($S_{i}$) and infected ($I_{i}$) compartments, each connected to the next compartment above. Discretised versions of the following continuous (in time and age) partial differential equations were used to describe the flow from one compartment to another in the computer simulation,

$\frac{\partial S_{i}(a,t)}{\partial t}+\frac{\partial S_{i}(a,t)}{\partial a}=-\beta S_{i}(a,t)\int_{a'} \frac{w(a,a')\sum_{j} \rho_{j}I_{j}(a',t)}{\sum_{j} I_{j}(a',t)+S_{j}(a',t)}da'-\mu(a)S_{i}(a,t)+\upsilon_{i-1}I_{i-1}(a,t)$ (1)

$\frac{\partial I_{i}(a,t)}{\partial t}+\frac{\partial I_{i}(a,t)}{\partial a}=\beta S_{i}(a,t)\int_{a'} \frac{w(a,a')\sum_{j} \rho_{j}I_{j}(a',t)}{\sum_{j} I_{j}(a',t)+S_{j}(a',t)}da'-\mu(a)I_{i}(a,t)-\upsilon_{i}I_{i}(a,t)$ (2)

$S_{i}$ and $I_{i}$ are functions of age *a* and time *t*;$\beta$ is the transmission parameter from infected to susceptible states and was estimated from fitting the model by maximum likelihood to the data; $w(a,a')$is a mixing matrix containing the information on the rate of mixing between individuals of age $a$ and $a'$ [1],

$\left(w(a,a')=\varepsilon\delta_{a,a'}+(1-\varepsilon)\frac{N_{a'}}{\sum_{a'} N_{a'}}\right)$, (3)

$\delta_{a,a'}$ is the Kronecker Delta[2]; $N_{a'}$ is the number of individuals of age $a'$ and $\varepsilon$ is a parameter that determines the population mixing patterns and it ranges from 0 (representing random mixing among age groups) to 1 (representing assortative mixing, i.e., each age group mixed only with itself); it is set to 0.5 in the model which represents an intermediate level of randomness and assortativity [3]; $\rho_{j}$ is the infectivity of infected individuals in compartment $I_{j}$; $\mu(a)$ is the death-rate form individuals of age *a* and the death-rates used here corresponded with the 2001 WHO Tanzanian and Gambian life tables; and $\upsilon_{i}$ is the recovery rate from infection for those in compartment $I_{i}$.

Recovery rate

The per individual rate of recovery, denoted by$\upsilon_{i}$(measured as the rate per year), from infection *i*, is assumed to change as an exponential function of *i* that begins at a rate $\upsilon_{1}$ (for recovery from first infection) and rises to a maximum rate $\upsilon_{\infty}$ (for recovery from a large number of infections),

$\begin{matrix} \\ \upsilon_{i}=\left(\upsilon_{1}-\upsilon_{\infty}\right) \exp\left[-\gamma\left(i-1\right) \right]+\upsilon_{\infty} \end{matrix}$. (4)

The parameters of this exponential function, $\upsilon_{1}$, $\upsilon_{\infty}$, and $\gamma$ were estimated by fitting (by maximum likelihood estimation) the model to the mean duration of infection for different age groups using published data[4] which have been recently re-analyzed [5]. The results of these parameter estimations are shown in Figure 3a and Table 2 ($\gamma$ is a rate constant that describes the rate of change of the recovery rate per infection).

Infectivity

We assume the infectivity of an individual, denoted by$\rho_{i}$, to be proportional to their bacterial load $l_{i}$, which we suggest is a function of the number of previous infections experienced by each individual in the population, a trend that is in agreement with the data from trachoma endemic communities in which the bacterial load decreases with age [6-9]. To capture this decreasing load with the number of previous infections, we use an exponential function such that the load declines from its initial value $\begin{matrix} \\ l_{1} \end{matrix}$ as described below,

$\begin{matrix} \\ l_{i}=l_{1}\exp\left[-\varphi\left(i-1\right)\right] \end{matrix}$ (5)

Parameter $\varphi$ can be estimated from age-stratified data on bacterial load by fitting equation (5) to the hyperendemic data (assumed to be at equilibrium) of West et al. [8]. The values obtained through maximizing the LL function are shown in Table 2 and the resulting curve of infectivity with age is shown in Figure 3b.

Parameter estimation

All of the parameters of the model ($\beta$, $\upsilon_{1}$, $\upsilon_{\infty}$, $\gamma$ and $\varphi$ (see Table 2)) were estimated at the same time in the hyperendemic case by maximizing the log-likelihood (LL) of the data on the age-dependent prevalence of infection, the recovery rate and the infection load. The LL function was constructed by assuming that the data for prevalence, infection load and infection duration were, respectively, binomially, Poisson, and exponentially distributed. The expression for the LL for the prevalence data is, therefore:

$LL_{prevalence}=\sum_{i} x_{i}\ln p_{i}+(n_{i}-x_{i})\ln(1-p_{i})$ (S1)

where$p_{i}$ is the model-generated infection prevalence for the age group *i*;$x_{i}$ and $n_{i}$ are the number of individuals infected and the total number of individuals in the data set for age group *i*. Using the central limit theorem, and ignoring additive constants, the corresponding LL expression for the infection load and duration data is:

$LL_{load/duration}=\sum_{i} \ln(\sqrt{Var(z_{i})})-\frac{(z_{i}-E(z_{i}))^{2}}{2Var(z_{i})}$ (S2)

where $z_{i}$ is the value, from the data-set, of the infection load or infection duration for age group *i*; $Var(z_{i})$ and $E(z_{i})$are the variance and expected value of the model-generated infection load or duration which are found assuming a Poisson distribution for the infection load data (for which the variance and mean are equal) and an exponential distribution for the duration of infection (for which the variance is the square of the mean). The six parameters were estimated by maximizing a LL expression that was the sum of the three individual expressions, which allowed the overall probability of the model generating the data to be maximised.

$LL_{total}=LL_{prevalence}+LL_{load}+LL_{duration}$ (S3)

Note that this expression assumes each likelihood term is independent of the others; since infection loads were measured only for those positive for infection, the prevalence and infection data were not strictly independent. However, individuals who were negative for infection would be expected to harbour a zero or very small infection load and would contribute minimally to the overall load, thereby effectively decoupling these measurements.

For the hypo- and mesoendemic cases, the transmission parameter $\beta$ (as the only free parameter) was estimated by maximum likelihood fitted to the infection prevalence data by formulating a negative LL expression identical to Equation (S1) (It was assumed that the values of the other four parameters remained the same as for the hyperendemic case). In addition to the maximum likelihood estimate of the parameter values, 95% confidence intervals were found by calculating profile log-likelihoods—for each of the parameters in the hyperendemic case and the parameter $\beta$ for the hypo- and mesoendemic cases—and assuming that this log-likelihood is chi-squared distributed about the maximum value [10].

Alternative model fit to the hyper-endemic community data

The data published by West et al. [8] for the prevalence of infection in Kongwa district, Tanzania, were used to fit the model as outlined in the main text and above. However, we performed a second illustrative model-fit in this study by considering that the number of individuals measured as positive for infection in each age group was overestimated in the original study by a factor of 52/36. This factor was obtained from the infection prevalence of the same community used by Blake et al. [11] (36%) (obtained from only those individuals with a positive infection load by quantitative PCR) and that quoted in the original study of West et al. [8] (52%) (obtained through qualitative PCR). This adjustment factor reduces each prevalence level along the entire age-dependent curve by an overall factor and results in a transmission parameter estimate of approximately 18 year^-1^ as opposed to 28 year^-1^ from the original fit. The second fit is illustrative here and the conclusions of the study apply to both model fits.

**References**

1. Anderson R, May R (1992) Infectious diseases of humans: dynamics and control: Oxford University Press. 768 p.

2. Boas ML (1983) Mathematical methods in the physical sciences. New York: John Wiley & Sons.

3. Gambhir M, Basanez MG, Turner F, Kumaresan J, Grassly NC (2007) Trachoma: transmission, infection, and control. Lancet Infect Dis 7: 420-427.

4. Bailey R, Duong T, Carpenter R, Whittle H, Mabey D (1999) The duration of human ocular *Chlamydia trachomatis* infection is age dependent. Epidemiol Infect 123: 479-486.

5. Grassly N, Ward ME, Ferris S, Mabey DC, Bailey RL (2008) The natural history of trachoma infection and disease in a Gambian cohort with frequent follow-up. PLoS Neglected Tropical Diseases (under review).

6. Solomon AW, Holland MJ, Burton MJ, West SK, Alexander ND, et al. (2003) Strategies for control of trachoma: observational study with quantitative PCR. Lancet 362: 198-204.

7. Solomon AW, Holland MJ, Alexander ND, Massae PA, Aguirre A, et al. (2004) Mass treatment with single-dose azithromycin for trachoma. N Engl J Med 351: 1962-1971.

8. West ES, Munoz B, Mkocha H, Holland MJ, Aguirre A, et al. (2005) Mass treatment and the effect on the load of *Chlamydia trachomatis* infection in a trachoma-hyperendemic community. Invest Ophthalmol Vis Sci 46: 83-87.

9. Burton MJ, Holland MJ, Makalo P, Aryee EA, Alexander ND, et al. (2005) Re-emergence of *Chlamydia trachomatis* infection after mass antibiotic treatment of a trachoma-endemic Gambian community: a longitudinal study. Lancet 365: 1321-1328.

10. Bolker B (2008) Ecological Models and Data in R: Princeton University Press. 408 p.

11. Blake I, Burton MJ, Solomon AW, West S, Munoz B, et al. (2009) Estimating Household and Community Transmission of Ocular Chlamydia trachomatis. PLoS NTDs 3: e401.
